# Predissociation Dynamics of Br_2_ in the [^2^Π_1/2_]_c_5d; 0_g_^+^ and [^2^Π_3/2_]_c_6d; 0_g_^+^ Rydberg States by Velocity Map Imaging Study

**DOI:** 10.1021/acsomega.2c02921

**Published:** 2022-08-11

**Authors:** Shoma Hoshino, Kento Ishii, Koichi Tsukiyama

**Affiliations:** Department of Chemistry, Faculty of Science Division I, Tokyo University of Science, 1−3 Kagurazaka, Shinjuku, Tokyo 184-8501, Japan

## Abstract

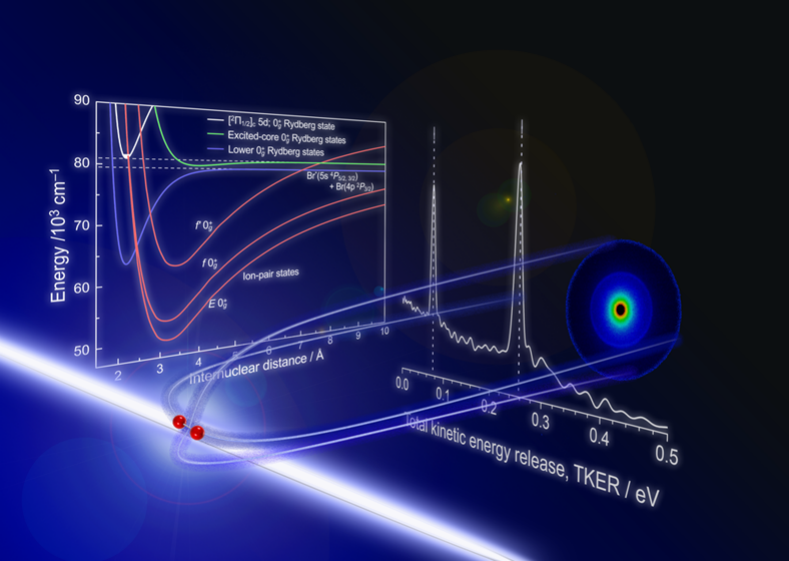

We investigated the predissociation dynamics from the [^2^Π_1/2_]_c_5d; 0_g_^+^ and [^2^Π_3/2_]_c_6d; 0_g_^+^ Rydberg states of Br_2_ using the velocity map imaging technique. Two-dimensional scattering images of the fragmented Br^+^ exhibited an isotropic feature upon the excitation of these Rydberg states. Analysis of the total kinetic energy release suggested the existence of the predissociation pathways to the dissociation limits of Br(5s, ^4^P_3/2_) + Br(4p, ^2^P_3/2_) and Br(5s, ^4^P_5/2_) + Br(4p, ^2^P_3/2_) *via* the 0_g_^+^ ion-pair states that interact with the lower and/or excited-core Rydberg states lying at long internuclear distance regions thorough the avoided crossing.

## 1 Introduction

Since the widespread use of tunable dye lasers in the late 1970s, the development of powerful spectroscopic techniques, such as laser-induced fluorescence (LIF),^1^ coherent anti-Stokes Raman spectroscopy (CARS),^2^ and resonance-enhanced multiphoton ionization (REMPI),^3^ has enabled us to investigate in detail the quantum state distributions of the internal states (rotational, vibrational, and electronic) of a wide range of products, resulting in dramatic advances in reaction dynamics research. However, spectroscopic approaches, in other words, measurements on the frequency axis, have the disadvantage that information about the translational motion (recoil velocity and anisotropy) is difficult to obtain, which is often essential for understanding reaction dynamics.

The ion imaging technique has been developed by Chandler and Houston to comprehensively elucidate the reaction mechanism, including information such as rate distribution and anisotropy parameters.^4,5^ In contrast to spectroscopic methods, ion imaging has a tremendous advantage in which all of the information including two-dimensional (2D) images, velocities, and angular distributions associated with chemical reactions can be obtained.^6^ Since then, techniques concerning ion imaging methods have been developed for a long time. As a result, the resolution of the images has been greatly improved,^7^ and image processing and analysis methods have been well established.^8^ The ion imaging technique is now recognized to be of high value in the field of chemical reaction dynamics.

Diatomic molecules, such as halogen molecules, are considered benchmarks for the study of reaction dynamics in the excited states due to their simple structures and have been studied both experimentally and theoretically for many years. In particular, the direct photodissociation and predissociation dynamics of the lower-valence electronic states of halogens have long been the subject of intensive research.^9,10^ However, in the higher-energy region, where a number of highly excited states such as the Rydberg states exist, the density of states becomes high and the perturbations between these states are extremely complicated. In this energy region, there are many dark states with relatively short lifetimes due to the presence of the reaction processes such as predissociation and autoionization. For example, spectroscopic analysis of the nonfluorescent Rydberg states of the bromine molecule, Br_2_, has been performed by Donovan et al.^11^ They have analyzed the Rydberg series of ^79^Br_2_ using resonance-enhanced multiphoton ionization (REMPI) spectroscopy and reported the spectra by monitoring ^79^Br_2_^+^ ions and ^79^Br^+^ ions. However, the mechanism for production of Br^+^ has not been discussed. The results show that the ^79^Br^+^ signal is dominant in the Rydberg state with two-photon energy of around 76,000 cm^–1^, suggesting that dissociation occurs more efficiently than in the other low-energy Rydberg states. Since the dissociation limit of Br_2_ into Br*(5s, ^4^P_5/2_) + Br(4p, ^2^P_3/2_) is ∼79,331 cm^–1^ from the lowest rovibrational level of the *X*^1^Σ_g_^+^ ground state,^12^ the dissociation channel to that limit might be observed from electronic states located in this energy region.

Rapid expansion of computational resources in recent years has made it possible to construct an overall picture of the potential energy of relatively small molecules. In particular, high-precision quantum chemical calculations of valence excited states, including ion-pair states of halogen molecules with many electrons, have been extensively performed, and calculation results with an accuracy satisfactorily enough to reproduce experimental data have been reported.^13,14^ However, quantum chemical calculations for highly excited levels, such as Rydberg states, located in the high-energy region are still challenging. One of the reasons that hinder the development of calculations is the lack of experimental data to be compared with the calculation results. In this respect, experimental investigations on the electronic structure and reaction dynamics in the high-energy region would be also indispensable for the future improvement of theoretical techniques.

In the present work, the dissociation processes in the [^2^Π_1/2_]_c_5d; 0_g_^+^ and [^2^Π_3/2_]_c_6d; 0_g_^+^ Rydberg states at excitation energies of around 81,000 cm^–1^ were investigated using the velocity map imaging (VMI) method.

## 2 Experimental Section

**Figure 1:**
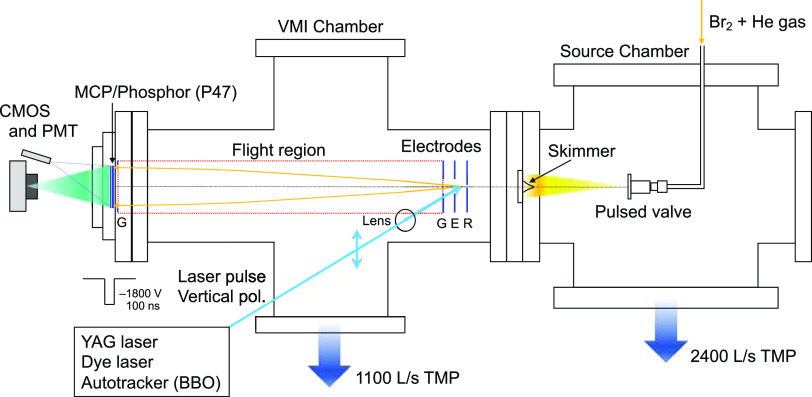
Overview of the experimental setup.

Experiments described in this work have been performed with an Eppink–Parker-type^7^ velocity map imaging (VMI) system. The schematic of the experimental setup is shown in Figure 1. The apparatus consists of two differentially pumped chambers, namely, for molecular beam source and ionization/VMI detection. The source chamber is evacuated by a 2400 L/s turbo molecular pump (Osaka Vacuum, TG2400F), while the ionization/VMI chamber is equipped with an 1100 L/s turbo molecular pump (Osaka Vacuum, TG1100F). Each turbo molecular pump was backed up by 35 L/s scroll pumps (Edwards, XDS35i). The source chamber was equipped with a pulsed valve (Parker-Hannifin, Series 9 with a 0.5 mm orifice) driven by an IOTA ONE controller. The ionization/VMI region is installed with a homemade linear time-of-flight mass (TOF-MS) spectrometer unit, which consisted of three stainless plates (repeller, extractor, and ground).

The Br_2_ vapor (∼2.3 kPa), purified in a vacuum condition, was diluted with He gas to prepare a gas mixture. The sample gas mixture at a stagnation pressure of ∼2 MPa was pulsed out into the chamber from a pulsed valve. Pressures in the chambers were maintained at ∼10^–3^ and 10^–5^ Pa in the source and ionization/VMI regions, respectively, during the measurements. The gas pulses with a duration of 190–200 μs were then collimated by a conical skimmer (Beam Dynamics, 1.0 mm orifice) located ∼15 cm from the valve. After ∼7 cm travel from the skimmer, the beams interacted with a laser pulse and photoionized between a pair of electrodes.

In this experiment, a single UV wavelength served as both two-photon excitation to the Rydberg states and subsequent one-photon ionization of the fragments. A frequency-doubled output, by an auto tracking system with a BBO crystal (Inrad Optics, Autotracker III), of the dye lasers (Continuum, ND6000) pumped by the third harmonic output of a *Q*-switched Nd^3+^:YAG laser (Continuum, Surelite II) operating at 10 Hz was used as the excitation/ionization light source. The UV laser power and pulse width were typically ∼0.05 mJ/pulse and ∼7 ns, respectively. UV laser pulse was focused by an *f* = 170 mm lens and introduced at the interaction region between the repeller and extractor electrodes in the VMI chamber.

The product ions are collinearly accelerated by a DC electric field into the 45 cm long flight region and detected by a chevron-type 42 mm imaging MCP detector (Hamamatsu Photonics, F2225 with P47 phosphor screen). Using a timed voltage pulse (∼100 ns) with a homemade high-voltage switch, the gain of the front MCP was gated to select ions of one particular mass, thus yielding mass-selective images. The 2D images on the phosphor screen are recorded by a CMOS camera (Hamamatsu Photonics, ORCA-spark) and stored in a PC using image acquisition and accumulation software (Hamamatsu Photonics, HiPic 9). Camera frames were accumulated for 60,000 shots to form a scattering image. Corrections for the radial distribution and translational energy of the fragment ions were performed by acquiring a photodissociation image of O_2_ at around 225.655 nm.^15^

## 3 Results and Discussion

### 3.1 Excitation of the Rydberg States of Br_2_

**Figure 2:**
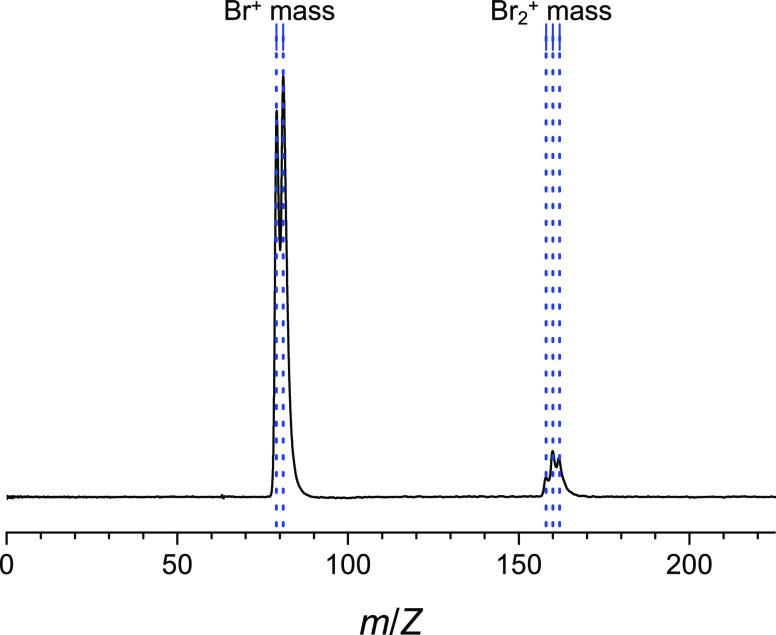
Time-of-flight mass spectrum obtained when the two-photon laser energy was adjusted to be ∼81,405 cm^–1^.

Rydberg states of Br_2_ were excited from the *X*^1^Σ_g_^+^ ground state by one-color two-photon absorption. Figure 2 shows the mass spectrum obtained when the two-photon wavenumber was adjusted to be ∼81,405 cm^–1^. The mass peak group at around *m*/*z* = 160 was attributed to Br_2_^+^, which has three isotopes, ^79^Br_2_, ^79^Br^81^Br, and ^81^Br_2_. Since the reduced masses of these isotopes are almost the same, the transitions of ^79^Br^81^Br and ^81^Br_2_ overlap with those of ^79^Br_2_ within the laser linewidth. The mass peak group observed at around *m*/*z* = 80 is attributed to the Br^+^ signal.

**Figure 3:**
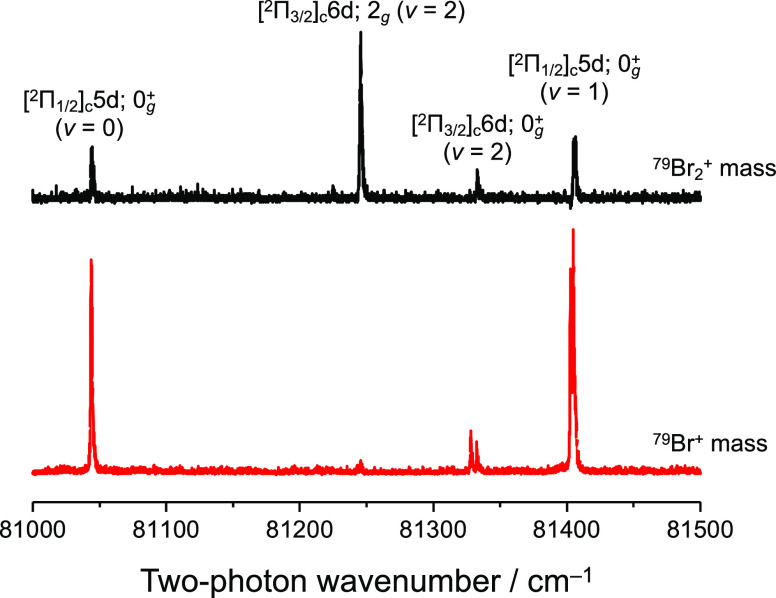
(2 + 1) REMPI spectra. The upper and lower parts were obtained by monitoring the ^79^Br_2_^+^ and ^79^Br^+^ mass channels, respectively.

Figure 3 shows the (2 + 1) REMPI spectra, where the upper and lower traces are obtained by monitoring ^79^Br_2_^+^ and ^79^Br^+^ mass channels, respectively. Four peaks are observed in the 81,000–81,500 cm^–1^ region, which are assigned to the transition to the [^2^Π_1/2_]_c_5d; 0_g_^+^ (ν = 0), [^2^Π_3/2_]_c_6d; 2_g_ (ν = 2), [^2^Π_3/2_]_c_6d; 0_g_^+^ (ν = 2), and [^2^Π_1/2_]_c_5d; 0_g_^+^ (ν = 1) Rydberg states from the lower wavenumber side.^11^ The Br_2_^+^ cation is undoubtedly generated from the subsequent one-photon absorption after two-photon excitation to the Rydberg state from the *X*^1^Σ_g_^+^ ground state, *i*.*e*.,
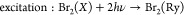
1
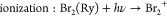
2

Though the intensity ratios differ, the fact that a similar REMPI spectrum with the same transition wavenumbers is recorded when the ^79^Br^+^ mass channel was monitored indicates that the dissociation of Br_2_ is occurring within the laser pulse width in the Rydberg states. Although there are several possible processes for the formation of Br^+^, the following process is most likely in this study.
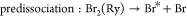
3
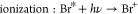
4

### 3.2 Velocity Map Images

**Figure 4:**
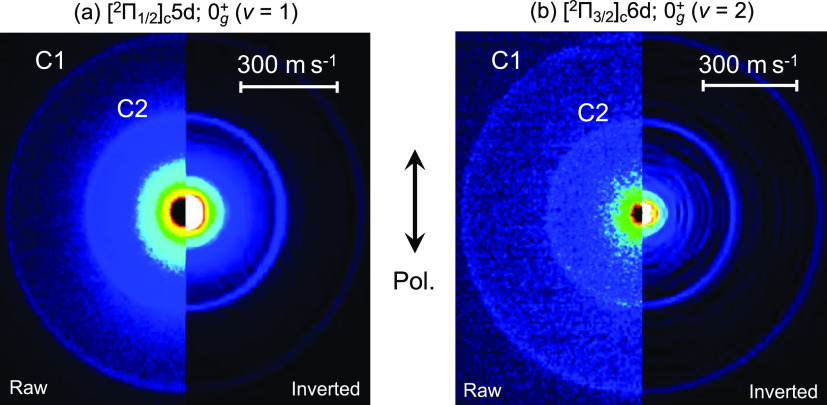
Fragmented Br^+^ ion images obtained by the excitation to (a) [^2^Π_1/2_]_c_5d; 0_g_^+^ (ν = 1) and (b) [^2^Π_3/2_]_c_6d; 0_g_^+^ (ν = 2) Rydberg states. The left side of each image is the raw image, and the right side is the Abel-inverted image reconstructed by the pBASEX method.

**Figure 5:**
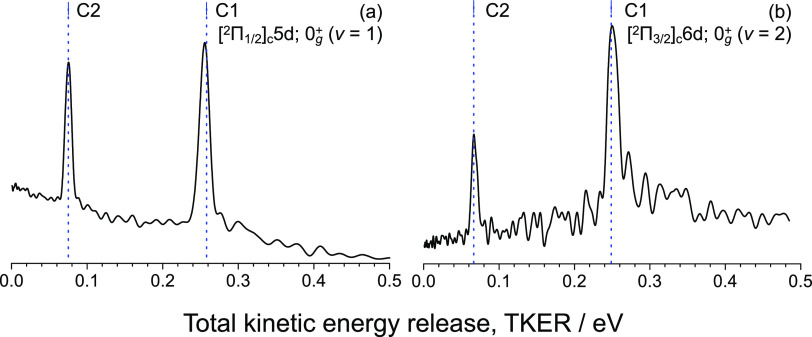
Distributions of the total kinetic energy release of fragments from the (a) [^2^Π_1/2_]_c_5d; 0_g_^+^ (ν = 1) and (b) [^2^Π_3/2_]_c_6d; 0_g_^+^ (ν = 2) Rydberg states. The blue dashed drop line represents the theoretical TKER predicted from eq 5 for the dissociation pathways of schemes (a) and (b).

The scattering image of ^79^Br^+^ was acquired by applying a fast, high-voltage pulse, ∼100 ns, which is much shorter than the difference between ^79^Br^+^ and ^81^Br^+^ in the arrival time to the MCP. As mentioned before, the transition wavenumbers of the three isotopes of Br_2_ are very close to each other. At the transition wavenumbers used in this study, the excitation of ^79^Br_2_ is dominant with simultaneous minor contributions from ^79^Br^81^Br and ^81^Br_2_. Therefore, the ion image obtained here is not only due to the dissociated fragment from pure ^79^Br_2_, although the interference of minor species is not critical enough to hamper the derivation of parameters such as translational energy. Figure 4a,b shows the fragmented Br^+^ ion images obtained by the excitation to the [^2^Π_1/2_]_c_5d; 0_g_^+^ (ν = 1) and [^2^Π_3/2_]_c_6d; 0_g_^+^ (ν = 2) Rydberg states, respectively. The left side of each picture is the raw image, while the right side is the Abel-inverted image reconstructed by the polar basis function expansion (pBASEX) method.^16^ A broad signal was observed at the center of the image in Figure 4. This signal centered at TKER ≈ 0 is thought to originate from the dissociation of (Br_2_)_*n*_ clusters possibly produced in a supersonic jet, which is not the main subject of this paper. In both Rydberg states, isotropic rings (anisotropy parameter β ≈ 0) with two different kinetic energies were observed (C1 and C2 in Figure 4). The radial intensity distributions were determined by integrating the obtained images over the entire angle, as shown in Figure 5. The left side (a) of Figure 5 corresponds to the [^2^Π_1/2_]_c_5d; 0_g_^+^ (ν = 1) state, and the right side (b) corresponds to the [^2^Π_3/2_]_c_6d; 0_g_^+^ (ν = 2) state. The solid black traces correspond to the experimental total kinetic energy release (TKER). The recoil energy was determined using the distance from the center of the image to the ring corresponding to each dissociation path and the time of flight for the detection of ^79^Br^+^ in the TOF-MS spectrum (Figure 2).

TKER can be determined using the two-photon energy used for excitation to the Rydberg state, the binding energy *D*_0_^diss^ (*X*),^17^ and internal energy *E*_int_ in the *X*^1^Σ_g_^+^ ground state of the Br_2_ molecule, and the energy *E*_el_ of the electronic state of the excited Br atom correlated to the dissociation limit as follows
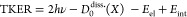
5

In this study, the internal energy *E*_int_ is assumed to be zero because the experiment was conducted under supersonic jet-cooled conditions. The rotational temperature of Br_2_ in the molecular beam produced in this experiment is about 25 K (see Section 3.3). The internal energy (rotational energy) at this temperature is estimated to be approximately 9 cm^–1^ (∼10^–3^ eV), which is negligibly small. Each of the two observed peaks can be well reproduced by the calculated values (vertical blue dashed lines) assuming the following dissociation and ionization pathways.^12^
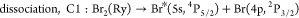
6a
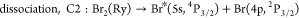
6b
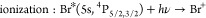
6c

**Figure 6:**
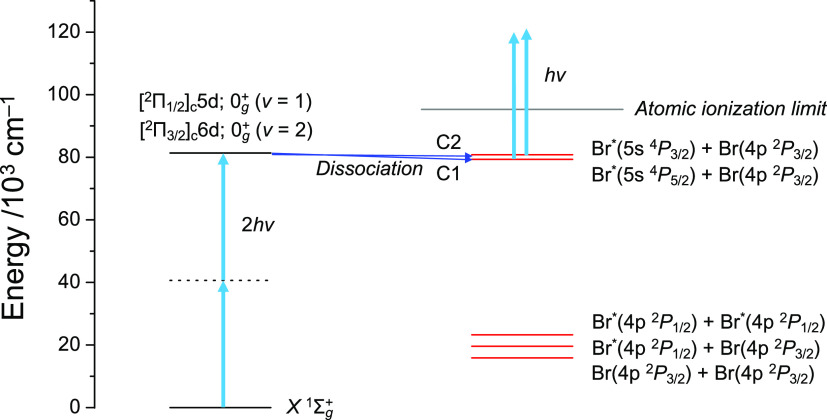
Diagram of the observed predissociation pathways from the [^2^Π_1/2_]_c_5d; 0_g_^+^ (ν = 1) and [^2^Π_3/2_]_c_6d; 0_g_^+^ (ν = 2) Rydberg states.

The calculated kinetic energy of the dissociated fragment from the [^2^Π_1/2_]_c_5d; 0_g_^+^ (ν = 1) state is 0.258 eV for Channel 1 and 0.0753 eV for Channel 2. For the [^2^Π_3/2_]_c_6d; 0_g_^+^ (ν = 2) state, TKERs of 0.249 and 0.0664 eV are expected for C1 and C2, respectively. The ionization energy of Br is *IE*(Br) = 95284.8 cm^–1^,^18^ and the energies of Br(5s, ^4^P_3/2_) and Br(5s, ^4^P_5/2_) from the ground state of Br(4p, ^2^P_3/2_) are 63436.5 and 64907.2 cm^–1^, respectively.^12^ The one-photon energies of the UV laser are 40702.5 cm^–1^ for the excitation to [^2^Π_1/2_]_c_5d; 0_g_^+^, (ν = 1) and 40666.5 cm^–1^ for [^2^Π_3/2_]_c_6d; 0_g_^+^ (ν = 2), which are sufficient for one-photon ionization of the Br(5s) atoms. Figure 6 illustrates the dissociation scheme of Br_2_ excited to the [^2^Π_1/2_]_c_5d; 0_g_^+^ and [^2^Π_3/2_]_c_6d; 0_g_^+^ Rydberg states. The two dissociation paths identified in this study are energetically correlated to the lowest (5s, ^4^P_5/2_) and second-lowest (5s, ^4^P_3/2_) excited Rydberg configurations of Br atoms. The predissociation limits correlating the ground state (4p, ^2^P_3/2_) and its spin-orbit excited state (4p, ^2^P_1/2_) are located in much lower energy regions and the laser wavelength used in this study is insufficient for one-photon ionization from 4p, ^2^P_3/2_, and ^2^P_1/2_. Therefore, even if the dissociation pathways to the lower energy limits exist, it will not be observed in the ion images. However, since the repulsive walls of the valence states are independent of the [^2^Π_1/2_]_c_5d; 0_g_^+^ and [^2^Π_3/2_]_c_6d; 0_g_^+^ Rydberg states and there is no avoided crossing, such a dissociation process is unlikely to occur.

### 3.3 Mechanism of Predissociation from the Rydberg States of Br_2_

**Figure 7:**
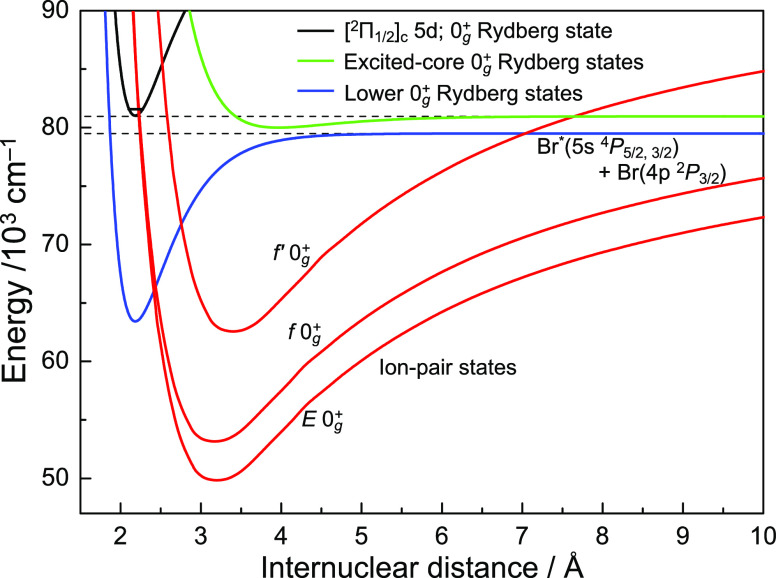
Schematic potential energy curves related to the predissociation pathways through the ion-pair state and lower and/or excited-core Rydberg states. The Br_2_ molecule in the [^2^Π_1/2_]_c_5d; 0_g_^+^ and [^2^Π_3/2_]_c_6d; 0_g_^+^ Rydberg states passes through the avoided crossing and transfers to the ion-pair state. Then, the molecule dissociates after passing through the second avoided crossing with the lower or excited-core Rydberg states at the longer internuclear distances.

The [^2^Π_1/2_]_c_5d; 0_g_^+^ and [^2^Π_3/2_]_c_6d; 0_g_^+^ Rydberg states converge with the ground state of the ion core (*X*^2^Π_1/2, 3/2_ of Br_2_^+^) and exist in the region of shorter internuclear distances than the ground state of neutral Br_2_. The 5s Rydberg states correlated with the dissociation limit Br*(5s) + Br(4p) exist in the lower-energy region. In general, the potential energy curves of the Rydberg states are similar to each other, so in the region around the equilibrium internuclear distance, the interaction of these lower Rydberg states with the [^2^Π_1/2_]_c_5d; 0_g_^+^ and [^2^Π_3/2_]_c_6d; 0_g_^+^ Rydberg states seems to be negligible. However, the [^2^Π_1/2_]_c_5d; 0_g_^+^/[^2^Π_3/2_]_c_6d; 0_g_^+^ states may be connected with lower 0_g_^+^ Rydberg states by a relay of the 0_g_^+^ ion-pair states, as shown in Figure 7. It is known that the higher vibrational levels of the ion-pair states of halogen molecules interact with the Rydberg states at a shorter internuclear distance. Kalemos et al. reported several avoided crossings on the inner and outer part of the *E* 0_g_^+^ (^3^P_2_) ion-pair state of I_2_ by *ab initio* multireference configuration interaction methods.^19^ Although the interaction of the [^2^Π_1/2_]_c_5d; 0_g_^+^ and [^2^Π_3/2_]_c_6d; 0_g_^+^ Rydberg states with the ion-pair states of Br_2_ has not been studied so far, the mixing of the ion-pair and Rydberg states is a commonly observed phenomenon.^20^ In Figure 7, the potential of the [^2^Π_1/2_]_c_5d; 0_g_^+^ Rydberg state is drawn using the parameter of the *X*^2^Π_1/2_ state of Br_2_^+^ cation.^21^ Although there may be several Rydberg states correlated to Br*(5s) + Br(4p), only the 5s; 0_g_^+^ Rydberg state is illustrated with a blue curve to avoid complexity. Br_2_ has four 0_g_^+^ ion-pair states: *E* 0_g_^+^ (^3^P_2_), *f* 0_g_^+^ (^3^P_0_), *f*′ 0_g_^+^ (^1^D_2_), and 0_g_^+^ (^1^S_0_). Among them, the 0_g_^+^ (^1^S_0_) state has not been observed experimentally while *ab initio* calculation estimates its location in an energy region about 79,000 cm^–1^ above the *X*^1^Σ_g_^+^ ground state.^22^ Since the ion-pair states have a longer internuclear distance than the Rydberg states, the interaction of the [^2^Π_1/2_]_c_5d; 0_g_^+^/[^2^Π_3/2_]_c_6d; 0_g_^+^ states with the 0_g_^+^ (^1^S_0_) state is unlikely. The potential minima of the *E* 0_g_^+^ (^3^P_2_), *f* 0_g_^+^ (^3^P_0_), and *f*′ 0_g_^+^ (^1^D_2_) states are located at ∼49,777.9, ∼53,101.7, and ∼65,512.0 cm^–1^ from the ground state, respectively, which are lower than the energy region surveyed in this study. The highly vibrational levels of these ion-pair states have not been experimentally investigated, and an accurate potential energy curve cannot be drawn over a wide range of internuclear distances. Therefore, in the potential energy curves shown in Figure 7, the RKR potential was adopted for the experimentally investigated region (near the potential minimum) and the inner potential wall was adjusted to reproduce the RKR potential in the low-energy region based on the results of *ab initio* calculation.^22^ Since the potential energy curves of the ion-pair states have similar shapes to each other, we referred to the known parameters of the *D* 0_u_^+^ (^3^P_2_) state of Br_2_ for the long-range Coulomb potential dominating the outer branch of the ion-pair potential energy curves.^23^ As a result, the *E* 0_g_^+^ (^3^P_2_) state intersects the asymptotes to the dissociation limits observed in the current experiment, Br*(5s, ^4^P_5/2, 3/2_) + Br(4p, ^2^P_3/2_), at ∼38 and ∼26 Å, respectively. The *f* 0_g_^+^ (^3^P_0_) states intersect the asymptote at ∼18 and ∼15 Å and at ∼8 and ∼7 Å for the *f*′ 0_g_^+^ (^1^D_2_) state, respectively.

On the other hand, it is known that halogen molecules have shallow Rydberg states (excited-core Rydberg states) in the long internuclear distance region. For example, the [^2^Σ_u,1/2_^+^]_c_6s; 0_u_^+^ and [^4^Σ_u,1/2_^–^]_c_6s; 0_u_^+^ states of the I_2_ molecule, correlated with the I(^2^P_3/2_) + I*(6s, ^4^P_3/2_) and I(^2^P_3/2_) + I*(6s, ^4^P_5/2_) dissociation limits, respectively, have been identified as an excited-core Rydberg state by the optical-triple resonance spectroscopy *via* the ion-pair states.^24,25^ Although no spectroscopic analysis of such shallow Rydberg states is currently available for the Br_2_ molecule, the shallow excited-core 0_g_^+^ Rydberg states may exist in the same energy region as the [^2^Π_1/2_]_c_5d; 0_g_^+^ and [^2^Π_3/2_]_c_6d; 0_g_^+^ Rydberg states. The energetic positional relationship and structure of the electronic state of Br_2_ are very similar to those of I_2_. For various valence, Rydberg, and ion-pair states, the I_2_ potential agrees well with the Br_2_ potential if the energy is multiplied by a factor of 1.135 and the internuclear distance by a factor of 0.86 relative to the ground-state dissociation limit. The excited-core Rydberg state of Br_2_ in Figure 7 is drawn by modifying the potential curves reported for I_2_. While the Br_2_ molecule is most likely to dissociate *via* the Rydberg ion-pair perturbation at the inner wall as discussed earlier, the possibility of the ion-pair-excited-core Rydberg and/or ion-pair-lower Rydberg perturbation at the relatively longer internuclear distances may not be discriminated. There seems to be no significant difference in the intensity ratios of C1 and C2, C2/C1, of 0.45 ± 0.05 and 0.31 ± 0.21 for the [^2^Π_1/2_]_c_5d; 0_g_^+^ and [^2^Π_3/2_]_c_6d; 0_g_^+^ states, respectively. The value of C2/C1 may be correlated to the magnitude of the interactions between the intermediate ion-pair state and the excited-core states; similar intensity ratios for the [^2^Π_1/2_]_c_5d; 0_g_^+^ and [^2^Π_3/2_]_c_6d; 0_g_^+^ states may imply the presence of an identical dissociation mechanism.

Next, we discuss the anisotropy of the obtained scattering distribution. As mentioned above, two isotropic rings, corresponding to dissociation paths (6a) and (6b) with different kinetic energies, were observed clearly in the experimental images. Since the rotational constants of the [^2^Π_1/2_]_c_5d; 0_g_^+^ and [^2^Π_3/2_]_c_6d; 0_g_^+^ Rydberg states have not been reported, the rotational contours of the REMPI spectra were analyzed by the Pgopher program,^26^ assuming the same rotational constant as the [^2^Π_3/2_]_c_4d; 1_g_ Rydberg state.^27^ The rotational temperature was found to be about 25 K. Assuming Boltzmann distribution at this rotational temperature, the rotational quantum number *J*_max_, giving the maximum population, is estimated to be about 10. Thus, the rotational period of a molecule in the Rydberg states shall be presumed to be approximately 20 ps at *J* = 10. On the other hand, an obvious line broadening in the rotational contour of the REMPI spectrum was seen. The linewidth of the dye laser used in the experiment, determined from the measurement of the fringe pattern of a Fabry–Perot Étalon, was ∼0.15 cm^–1^ for the fundamental (visible) output. Considering that the linewidth increases by a factor of √2 upon second harmonics generation, the resolution of the spectrum is expected to be about 0.21 cm^–1^. Assuming this laser linewidth, the homogeneous width (lifetime width) estimated from the profile simulation is ∼0.5 cm^–1^, which corresponds to 10 ps for the lifetime of the excited states. Therefore, in the [^2^Π_1/2_]_c_5d; 0_g_^+^ and [^2^Π_3/2_]_c_6d; 0_g_^+^ Rydberg states, the predissociation process proceeds on a time scale comparable to or shorter than the rotational period. In such cases, the angular distribution of fragments is expected to show anisotropic behavior. In general, in photodissociation of diatomic molecules, should the molecule rotate before dissociation, the degree of correlation between the electric field vector of the light and the fragment recoil direction will be lowered but not completely to zero. The limiting values for anisotropy parameters for parallel and perpendicular transitions are β_||_ = 0.5 and β_⊥_ = −0.25, respectively.^8^ Therefore, the isotropy of the observed images in the present work cannot be interpreted from the perspective of the lifetime of the excited state. One possible explanation for the isotropic pattern in Figure 4 despite the short lifetime in the Rydberg state is that two-photon absorption occurs through the inner walls of various valence states lying in the one-photon energy region. Current experiments do not allow us to confirm whether the two-photon excitation to the Rydberg state occurs under nonresonance or *via* a sequence of two steps. In the latter case, Ω = 0_u_^+^ or 1_u_ valence states can behave as intermediate states. Dixon derived expressions for recoil anisotropy following multiphoton processes *via* near-resonant intermediate states.^28^ In ref (28), the disappearance of anisotropy in the final state distribution due to multiple competing pathways is discussed as follows. The transition from the *X*^1^Σ_g_^+^ ground state to the 0_u_^+^ intermediate state has Σ → Σ-type anisotropy (cos^2^ θ), while the transition to the 1_u_ intermediate state has Σ → Π-type anisotropy (sin^2^ θ). According to his theoretical approach, the fragment image from the Rydberg state excited *via* a single Ω = 0_u_^+^ or 1_u_ valence state should show anisotropy. However, if they contribute with equal amplitude and sign, then the intermediate molecules produced by one-photon absorption are isotropic distributed (β = 0). Furthermore, if the Rydberg state is produced by one-photon absorption from that intermediate state, the distribution will be isotropic as well. *Ab initio* calculations^29^ have revealed the existence of repulsive walls of various valence states of Br_2_ in the one-photon energy region in this experiment, and indeed, the corresponding weak absorption has been observed.^30^ The presence of such valence states may be responsible for the isotropy of fragments produced by the predissociation in the Rydberg state. In this study, we have not observed a direct dissociation process in the valence states following one-photon absorption, but dissociative fragments in the one-photon energy region have been observed upon Rydberg state excitation of the iodine molecule. A similar excitation/predissociation scheme may apply for the Rydberg state of Br_2_.^31,32^

Another possibility is the involvement of relatively long-lived (∼ns) ion-pair states in the dissociation pathway. It is possible that dissociating molecules are trapped in the bound state for longer than the rotational period, resulting in the loss of anisotropy of the final fragments.

## 4 Conclusions

In this study, velocity map imaging of the charged photofragments technique was applied to study the predissociation dynamics in the [^2^Π_1/2_]_c_5d; 0_g_^+^ and [^2^Π_3/2_]_c_6d; 0_g_^+^ Rydberg states of Br_2_. For each Rydberg state, two isotropic fragment images with different kinetic energies were observed. Analysis of the distribution of the total kinetic energy release indicates that these two dissociation paths originate from the predissociation paths of Br_2_(Ry) → Br*(5s, ^4^P_5/2_) + Br(4p, ^2^P_3/2_) and Br_2_(Ry) → Br*(5s, ^4^P_3/2_) + Br(4p, ^2^P_3/2_). These dissociation processes are thought to be mediated by the 0_g_^+^ ion-pair and lower and/or excited-core Rydberg states, which are expected to exist at longer internuclear distances. According to the analysis of REMPI spectra, the lifetime of the excited Rydberg state is about 10 ps, which is comparable to or shorter than the rotational period of the excited molecule. It can be interpreted that two-photon excitation is a sequential process *via* repulsive walls of various valence states, and the anisotropy is averaged out, resulting in an isotropic fragment image. The analysis of perturbations between the ion-pair states and the Rydberg states and collaboration with theoretical researchers will be necessary to further investigate the predissociation dynamics of the Rydberg states of Br_2_.

## References

[ref1] Zare R. N.; Dagdigian P. J. Tunable Laser Fluorescence Method for Product State Analysis: Laser-induced fluorescence is used as a detector of collisionally unrelaxed products of chemical reactions. Science 1974, 185, 739–747. 10.1126/science.185.4153.739. 4843375

[ref2] Valentini J. J. Spectrometric Techniques; Vanasse G. A., Ed.; Academic Press: London, 1983; Vol. IV, pp 1–62. 10.1016/B978-0-12-710404-1.50007-3

[ref3] Ashfold M.N.R.; Howe J. D. Multiphoton Spectroscopy of Molecular Species. Annu. Rev. Phys. Chem. 1994, 45, 57–82. 10.1146/annurev.pc.45.100194.000421.

[ref4] Heck A.J.R.; Chandler D. W. Imaging Techniques for the Study of Chemical Reaction Dynamics. Annu. Rev. Phys. Chem. 1995, 46, 335–372. 10.1146/annurev.pc.46.100195.002003. 24329711

[ref5] Houston P. L. Snapshots of Chemistry: Product Imaging of Molecular Reactions. Acc. Chem. Res. 1995, 28, 453–460. 10.1021/ar00059a003.

[ref6] Chandler D. W.; Houston P. L. Two-dimensional imaging of state-selected photodissociation products detected by multiphoton ionization. J. Chem. Phys. 1987, 87, 1445–1447. 10.1063/1.453276.

[ref7] Eppink A.T.J.B.; Parker D. H. Velocity map imaging of ions and electrons using electrostatic lenses: Application in photoelectron and photofragment ion imaging of molecular oxygen. Rev. Sci. Instrum. 1997, 68, 3477–3484. 10.1063/1.1148310.

[ref8] Imaging in Molecular Dynamics; Whitaker B. J., Ed.; Cambridge University Press: Cambridge, 2003. 10.1017/CBO9780511535437

[ref9] Vallance C. Multi-mass velocity-map imaging studies of photoinduced and electron-induced chemistry. Chem. Commun. 2019, 55, 6336–6352. 10.1039/C9CC02426C. 31099379

[ref10] Asano Y.; Yabushita S. Theoretical study on the nonadiabatic transitions in the photodissociation of Cl_2_, Br_2_, and I_2_. Bull. Korean Chem. Soc. 2003, 24, 703–711. 10.5012/bkcs.2003.24.6.703.

[ref11] Donovan R. J.; Flexen A. C.; Lawley K. P.; Ridley T. The (2+1) REMPI spectroscopy of jet-cooled Br_2_. Chem. Phys. 1998, 226, 217–228. 10.1016/S0301-0104(97)00324-8.

[ref12] Alexander K. NIST Atomic Spectra Database - SRD 78. 10.18434/T4W30F

[ref13] Alekseeva S. V.; Alekseev V. A. Ab Initio Study of Ion-Pair States of Halogen Molecules. Russ. J. Phys. Chem. 2020, 94, 1382–1395. 10.1134/S0036024420070043.

[ref14] Hoshino S.; Alekseev V. A.; Ishii K.; Tsukiyama K. Electronic transition dipole moment function of the *f′* 0^+^ (^1^*D*_2_) – *X*^1^Σ^+^ transition of ICl. J. Quant. Spectrosc. Radiat. Transfer 2022, 277, 107992 10.1016/j.jqsrt.2021.107992.

[ref15] Parker D. H.; Eppink A.T.J.B. Photoelectron and photofragment velocity map imaging of state-selected molecular oxygen dissociation/ionization dynamics. J. Chem. Phys. 1997, 107, 2357–2362. 10.1063/1.474624.

[ref16] Garcia G. A.; Nahon L.; Powis I.; et al. Two-dimensional charged particle image inversion using a polar basis function expansion. Rev. Sci. Instrum. 2004, 75, 4989–4996. 10.1063/1.1807578.

[ref17] Focsa C.; Li H.; Bernath P. F. Characterization of the Ground State of Br_2_ by Laser-Induced Fluorescence Fourier Transform Spectroscopy of the *B*^3^Π_0*u*_^+^ – *X*^1^Σ_*g*_^+^ System. J. Mol. Spectrosc. 2000, 200, 104–119. 10.1006/jmsp.1999.8039. 10662581

[ref18] Ionization Potentials of Atoms and Atomic Ions, Handbook of Chemistry and Physics, Lide D. R. 1992, pp 10–211.

[ref19] Kalemos A.; Valdeś Á.; Prosmiti R. An ab Initio Study of the *E*^3^Π_*g*_ State of the Iodine Molecule. J. Phys. Chem. A 2012, 116, 2366–2370. 10.1021/jp3000202. 22295971

[ref20] Donovan R. J.; Lawley K. P.; Ridley T. A heavy Rydberg quantum defect analysis of the perturbed *D*(0_*u*_^+^) ion-pair state of Br_2_. Chem. Phys. 2015, 463, 145–148. 10.1016/j.chemphys.2015.10.015.

[ref21] Harris T.; Eland J.H.D.; Tuckett R. P. The *A*-*X* system of Br_2_^+^ radical cations. J. Mol. Spectrosc. 1983, 98, 269–281. 10.1016/0022-2852(83)90243-6.

[ref22] Ovchinnikova N. E.; Alekseev V. A. An Ab Initio Study of Ion-Pair States of Br_2_ Molecule. Opt. Spectrosc. 2016, 120, 192–198. 10.1134/S0030400X16020181.

[ref23] Lipson R. H.; Hoy A. R. VUV Laser Spectroscopy of Ion-Pair States of Br_2_. J. Mol. Spectrosc. 1989, 134, 183–198. 10.1016/0022-2852(89)90141-0.

[ref24] Sjödin A. M.; Ridley T.; Lawley K. P.; Donovan R. J. Observation of a new high-energy, shallow-bound Rydberg state in I_2_ by optical triple resonance. Chem. Phys. Lett. 2015, 412, 110–115. 10.1016/j.cplett.2005.06.095.

[ref25] Sjödin A. M.; Ridley T.; Lawley K. P.; Donovan R. J. Observation of a substantially-bound excited-core Rydberg state in I_2_ by optical triple resonance. Chem. Phys. Lett. 2005, 416, 64–69. 10.1016/j.cplett.2005.09.029.

[ref26] Western C. M. PGOPHER: A program for simulating rotational, vibrational and electronic spectra. J. Quant. Spectrosc. Radiat. Transfer 2017, 186, 221–242. 10.1016/j.jqsrt.2016.04.010.

[ref27] Xu L.; Wang Y.; Wang P.; Li F. Rotational analyses and angular momentum assignments of [^2^Π_3/2_]4*d* Rydberg states of Br_2_. J. Chem. Phys. 1994, 101, 3524–3530. 10.1063/1.467538.

[ref28] Dixon R. N. Recoil anisotropy following multiphoton dissociation via near-resonant intermediate states. J. Chem. Phys. 2005, 122, 194302 10.1063/1.1896951. 16161568

[ref29] da Silva Gomes J.; Gargano R.; Martins J. B.; de Macedo L.G.M. Relativistic Four-Component Potential Energy Curves for the Lowest 23 Covalent States of Molecular Bromine (Br_2_). J. Phys. Chem. A 2014, 118, 5818–5832. 10.1021/jp4114283. 24779448

[ref30] Tellinghuisen J. Equilibrium constants from spectrophotometric data: dimer formation in gaseous Br_2_. J. Phys. Chem. A 2008, 112, 5902–5907. 10.1021/jp8020358. 18540663

[ref31] Bogomolov A. S.; Grüner B.; Kochubei S. A.; Mudrich M.; Baklanov A. V. Predissociation of high-lying Rydberg states of molecular iodine via ion-pair states. J. Chem. Phys. 2014, 140, 124311 10.1063/1.4869205. 24697445

[ref32] von Vangerow J.; Bogomolov A. S.; Dozmorov N. V.; Schomas D.; Stienkemeier F.; Baklanov A. V.; Mudrich M. Role of ion-pair states in the predissociation dynamics of Rydberg states of molecular iodine. Phys. Chem. Chem. Phys. 2016, 18, 18896–18904. 10.1039/C6CP02160C. 27353150

